# Altered acylated ghrelin response to food intake in congenital generalized lipodystrophy

**DOI:** 10.1371/journal.pone.0244667

**Published:** 2021-01-07

**Authors:** Camilla O. D. Araújo, Renan M. Montenegro, Amanda P. Pedroso, Virgínia O. Fernandes, Ana Paula D. R. Montenegro, Annelise B. de Carvalho, Lila M. Oyama, Carla S. C. Maia, Eliane B. Ribeiro

**Affiliations:** 1 Departamento de Fisiologia, Universidade Federal de São Paulo (UNIFESP), Escola Paulista de Medicina, São Paulo, São Paulo, Brazil; 2 Faculdade de Medicina Universidade Federal do Ceará, Departamento de Medicina Clínica e Departamento de Saúde Comunitária, Brazilian Group for the Study of Inherited and Acquired Lipodystrophies (BRAZLIPO) - Hospital Universitário Walter Cantídio, Fortaleza-Ceará, Brazil; 3 Departamento de Nutrição, Universidade Estadual do Ceará (UECE), Campus do Itaperi, Fortaleza, Ceará, Brazil; Medical University of Vienna, AUSTRIA

## Abstract

**Background:**

Patients with congenital generalized lipodystrophy (CGL) have very low levels of leptin and are described as having a voracious appetite. However, a direct comparison between CGL and eutrophic individuals is lacking, regarding both appetite parameters and acylated ghrelin, the hormone form that is active in acute food intake stimulation. The objective of the present study was to address whether and in what extent the subjective appetite parameters and acylated ghrelin response to a meal are affected in CGL individuals, in comparison to eutrophic individuals. Additionally, an obese group was included in the study, to allow the comparison between a leptin-resistant and a leptin-deficient condition on these aspects.

**Methods:**

Eutrophic controls (EUT, n = 10), obese subjects (OB, n = 10) and CGL (n = 11) were fasted overnight and then received an *ad libitum* meal. Blood was collected and the visual analogue scale was applied before and 90 minutes after the meal. An additional blood sample was collected at 60 minutes for ghrelin determination.

**Results:**

The CGL patients showed low fasting levels of leptin and adiponectin, dyslipidemia, and insulin resistance. The caloric intake was similar among the 3 groups. However, both CGL (p = 0.02) and OB (p = 0.04) had shorter satiation times than EUT. The CGL patients also had lower satiety time (p = 0.01) and their sensation of hunger was less attenuated by the meal (p = 0.03). Fasting acylated ghrelin levels were lower in CGL than in EUT (p = 0.003). After the meal, the levels tended to decrease in EUT but not in CGL and OB individuals.

**Conclusion:**

The data indicate that, although not hyperphagic, the CGL patients present appetite disturbances in relation to eutrophic individuals. Their low fasting levels of acylated ghrelin and the absence of the physiological drop after meal intake suggest a role of these disturbances in hunger attenuation and satiety but not in acute satiation.

## Introduction

Congenital generalized lipodystrophy (CGL), a disease first reported in two Brazilian brothers [1], is a rare (1/10 million) condition caused by autosomal recessive mutations that lead to an almost total absence of adipose tissue. The number of reported cases worldwide varies from 332 [2] to around one thousand [3], the majority coming from Brazil, Lebanon, and Scandinavia. In Brazil, the largest number of cases is found in the Rio Grande do Norte state, followed by the Ceará state [4–6].

Mutations in four different genes have been described in CGL patients (*AGPAT2*, *BSCL2*, *CAV1*, *PTRF*) which impair fat storage by affecting triglycerides and phospholipids synthesis, lipid droplet formation and endocytosis, or adipocyte differentiation, in different degrees. The metabolic consequences are severe and include hypertriglyceridemia, insulin resistance, diabetes mellitus, and liver steatosis. The very low adiposity leads to decreased levels of leptin and adiponectin [7–9]. However, data on the status of other adipokines in CGL are scarce. Increased levels of angiopoietin-like protein 3 have been reported in a group including patients with either CGL or acquired lipodystrophy [10]. Normal levels of epidermal fatty acid-binding protein and increased levels of growth differentiation factor 15 have been reported in patients with generalized lipodystrophies [11,12].

Both leptin and insulin have circulating levels proportional to body adiposity and exert important influence on energy homeostasis by signaling body energy status to the central nervous system, acting mainly at the hypothalamus. They inhibit food intake through inhibition of hypothalamic neurons expressing orexigenic mediators, such as neuropeptide Y (NPY) and *Agouti*-related peptide (AgRP), and stimulation of anorexigenic mediators such as proopiomelanocortin (POMC) and cocaine- and amphetamine- regulated transcript (CART) [13].

The gastric hormone ghrelin also interacts with hypothalamic neurons to modulate food intake but, unlike leptin and insulin, it exerts an orexigenic action and acts as an acute hunger stimulator. This hormone is present in the circulation in both a deacylated and an acylated form. The acylation process confers to the molecule the ability to interact with its specific hypothalamic receptors, mediators of the orexigenic effect [14]. The physiological role of the hormone as an appetite stimulator is compatible with its demonstrated acute variations during the fasting/meal cycle with increased levels in the pre-ingestion hours and a prominent fall after food intake [15,16]. Besides its central effects, *in vitro* experiments have shown that ghrelin inhibits lipolysis [17] and stimulates lipogenesis in adipocytes [18].

Data on the ghrelin levels in CGL are scarce. At the best of our knowledge, only one study addressed this topic in comparison to eutrophic individuals, but it evaluated only total ghrelin levels, having reported blunting of the expected post-meal decline in the two patients evaluated [19]. Interestingly, in obesity, in which a common condition is leptin-resistance, rather than leptin deficiency, a similar pattern has been described for acylated ghrelin [20,21]. Since acylated ghrelin is the hormone form most relevant to appetite stimulation, the lack of knowledge on its status in CGL warrants investigation.

Although high energy intake [11] and voracious appetite [22] have been described in CGL individuals, an evaluation of their acute ingestion in comparison to normal individuals is lacking. The visual analogue scale (VAS) has been used to investigate subjective sensations related to food intake, such as satiety and satiation [23]. Using this approach in groups of patients that included various types of lipodystrophy, it has been shown that the treatment with recombinant leptin decreased hunger and increased satiety [24–26]. However, a direct comparison of these aspects between individuals presenting CGL and eutrophic subjects has not yet been performed.

The objective of the present study was to address whether, and in what extent, the subjective appetite parameters and acylated ghrelin response to a meal are affected in CGL individuals, in comparison to eutrophic individuals. Additionally, an obese group was included in the study, to allow the comparison between a leptin-resistant and a leptin-deficient condition on these aspects.

## Methods

The present study was approved by the Ethics Committee of the Walter Cantídio Hospital (Fortaleza, CE, Brazil, CAAE: 51022215.5.0000.5045) and by the Research Ethics Committee of the Universidade Federal de São Paulo (São Paulo, SP, Brazil, CAAE: 14167219.0.0000.5505).

The participants with CGL were recruited among the 21 patients treated at the Walter Cantídio University Hospital (Ceará, Brazil), one of the two the reference centers for Inherited and Acquired Lipodystrophies in Brazil. During the period of the study, a total of around 66 alive patients with the diagnosis of CGL were known in Brazil (44 in Rio Grande do Norte state and 22 in Ceará state) [27]. The CGL patients were contacted during their routine consultations. In a pilot study, we observed that it would not be appropriate to include the patients younger than 7 years, due to their difficulty to understand the instructions pertaining the different tests. So, we included initially only the 14 older patients. Of these, two were excluded due to clinical conditions (1 with thyroid cancer and 1 with severe liver failure) and one did not accept to participate in the study. Of the 11 subjects with congenital generalized lipodystrophy (CGL group, 64% female/36% male), nine were tested for the *AGPAT2* and *BSCL2* mutations. *AGPAT2* mutations were detected in 4 patients and the *BSCL2* mutations were detected in 2 patients while they were absent in the other 3 patients. All patients had the clinical diagnosis of CGL [28]. Their demographic characteristics are shown in Table 1.

**Table 1:** Socio-demographic and physical activity characteristics of the CGL patients.

CGL (n = 11)		N (%)
Family income	< 1 minimum wage	7 (63.6)
	>1 ≤ 3 minimum wage	3 (27.4)
	>3 ≤ 6 minimum wage	1 (9.0)
Origin	Fortaleza (the capital of the State of Ceará)	6 (54.5)
	Inland cities	5 (45.5)
Schooling	Primary education	7 (63.6)
	High school	3 (27.4)
	University	1 (9.0)
Physical activity	Inactive	4 (36.5)
	Moderately active	6 (54.5)
	Active	1 (9.0)

Ten healthy eutrophic volunteers, with no familial connection with the patients, served as controls (EUT group). The EUT subjects and the CGL patients were paired by sex, age, and body mass index (BMI). Ten obese subjects (BMI = 30–39.9 kg/m^2^, OB group) with no familial connection with the CGL patients were included in the study in order to allow the comparison between a leptin-deficient and a leptin-resistant condition. The obese individuals were not under nutritional counseling and they were paired by gender and age to the EUT group. The subjects aged 18 years or more signed the written informed consent form while the person responsible for the individuals aged less than 18 years signed the written informed assent. The EUT and OB participants were recruited among the persons accompanying patients at the hospital.

The treatment received by the CGL patients consisted of endocrinological and nutritional assessments, aimed at controlling the metabolic alterations. All patients were under treatment with fibrates for hypertriglyceridemia and with metformin for diabetes. Additionally, the patients showing hypertension were receiving hydrochlorothiazide. No other medical conditions were present in the patients. The nutritional treatment consisted of a hypocaloric diet containing 50–60% of carbohydrates, 20–30% of fat, and 10–20% of protein. Additionally, physical exercise was advised [8]. The EUT subjects presented no clinical diseases and the OB individuals were not included if they were under treatment for diabetes.

### Study design

All tests were performed at the Clinical Research Unit of the Walter Cantídio University Hospital, between November 2017 and September 2018. The study was performed in two consecutive days. In the first day, starting at around 9 a.m., the individuals responded to the socioeconomic questionnaire and were submitted to anthropometric measurements. They were instructed to abstain from alcohol, caffeine and strenuous activities and to keep record of all foods ingested until the start of a 12-h fasting. They arrived at the hospital at 7 a.m. of the next day, responded to the food record questionnaire and to the visual analogue scale questionnaire (VAS), and had a catheter inserted in the brachial vein. The food record was conducted by a trained interviewer, following the automated multiple pass method (AMPM) [29].

A basal blood sample (T0) was collected and then they had access to a meal buffet *ad libitum*. Additional blood sample were collected at the times 60 (T60) and 90 (T90) minutes after the end of the meal. The VAS questionnaire was applied again at T90.

### Test meal

The test was conducted in a private room equipped with a white chair and table. Upon entering the room, the participants received the following instructions: you may eat everything you like, as much as you want, but you may not to eat anything you do not like. They were also said to eat until complete fullness and then leave the room immediately. The satiation time was considered as the period elapsed between the initiation of the meal and the leaving of the meal room. The satiety time was considered as the period elapsed between the leaving of the meal room and their reporting of desire to eat another meal [24].

The foods available in the meal consisted of 12 items habitually consumed by the subjects, according to the Brazilian Survey of Familial Budget [30] and as assessed by three food records performed in CGL patients only, in non-consecutive days (2 week days and one weekend day [29]) in the 4 months before the test. The meal included bread, tapioca, corn couscous, salty biscuit, sweet biscuit, cake without filling, apple, banana, ham, cheese, mortadella, margarine, orange juice, coffee, milk, coffee with milk, chocolate, milk flour and yogurt. The caloric density of approximately 7,000 kcal corresponded to around 3 times the measured caloric intake of the patients and contained 54.5% carbohydrates, 12.5% proteins and 34% lipids.

### Visual analogue scale (VAS)

This scale has been validated for the Portuguese language [31]. It consists of 8 questions about feelings of hunger, satiation, satiety, and prospective food consumption, as well as about the desire to eat specifically sweet, salty, fatty or tasty foods. The participants were instructed to score their feelings in a 100 mm-long line, in which a word expressing the weakest and the strongest feeling about each aspect was written in the left and right extremities, respectively. The segments were then measured. The appetite score was calculated as: “desire to eat + hunger + (150 –satiety) + prospective food consumption/4”. The desire to eat was calculated as the average of the reported desires to eat sweet, salty, fatty and tasty foods [23,32].

### Blood measurements

The analyses of lipid and glycemic profiles were performed by colorimetric methods, following the manufacturer’s instructions (Labtest Diagnóstica, Lagoa Santa, MG, Brazil).

ELISA kits (Millipore, Billerica, MA, USA) were used to determine the serum levels of insulin (sensitivity 2–20 μU/ml, intra-assay precision 9%, inter-assay precision 5.1%), leptin (sensitivity 31.25–2000 pg/ml, intra-assay precision 4.6%, inter-assay precision 8%), adiponectin (sensitivity 62.5–4000 pg/ml, intra-assay precision 3.9%, inter-assay precision 17.5%) and PAI-1 (sensitivity -20-0.3125 ng/ml, intra-assay precision 10%, inter-assay precision 4.2%) as well as the plasma levels of acylated ghrelin (sensitivity 36.3–1163 pg/ml, intra-assay precision 4.9%, inter-assay precision 9.5%) (Millipore). For acylated ghrelin, 100 μL of serine protease inhibitors (Pefabloc, Sigma-Aldrich, St Louis, MO, USA) was added to 1 ml of blood, before centrifugation and the plasma was acidified with HCl (1mol/l, 100μl/ml of plasma).

### Statistical analysis

The normality of the variables was tested by the Kolmogorov-Smirnov test and the homoscedasticity of variances was tested by the Levene’s test. For comparisons among the 3 groups (EUT, OB, and CGL), the parametric variables were expressed as Means ± SEM and compared by one-way ANOVA followed by the Tukey HSD test for multiple comparisons while the non-parametric variables were expressed as Median (minimum; maximum) and compared by the Kruskal-Wallis test followed by the Dunnett’s test. The comparisons, within each group, of ghrelin levels before and 60 minutes after meal intake, were performed by two-sided paired Student’s “t” test. Significance was set at p ≤ 0.05. The SPSS 22.0 software (IBM, Armonk, NY, USA) was used for these analyses.

## Results

The food record of the day before the test meal (Table 2), confirmed the three prior food records of the CGL patients (S1 Table). No significant differences between the CGL and the EUT subjects were found. PUFA intake was significantly higher in the CGL than in the OB group (Table 2).

**Table 2:** Clinical characteristics and food records of CGL patients, obese (OB) and eutrophic subjects (EUT).

	EUT (n = 10)	OB (n = 10)	CGL (n = 11)
**Clinical characteristics**			
**Age (years)**	19.6 ± 2.6	22.4 ± 3.1	21.5 ± 2.7
**Body mass index (kg/m^2^)**	20.5 ± 0.8	31.0 ± 1.5	21.7 ± 0.8
**Presence of SAH**	0	1	2
**Presence of DM**	0	0	8
**24-h food record**			
**Calories (kcal)**	1,818.8 ± 181.5	1,689.7 ± 257.8	1,885.6 ± 265.5
**Carbohydrate (kcal)**	809.9 ± 119.0	785.1 ± 154.7	1,018.0 ± 183.3
**Protein (kcal)**	402.9 ± 91.4	373.1 ± 46.4	517.3 ± 77.3
**Lipid (kcal)**	526.3 ± 83.2	591.2 ± 1332.	348.8 ± 61.6
**SFA (%)**	35.2 (15.3; 51.0)	38.3 (29.3; 105.0)	33.3 (27.7; 44.8)
**PUFA (%)**	18.4 ± 1.8	16.4 ± 1.8	23.0 ± 2.0 ^#^
**MUFA (%)**	30.7 (15.0; 58.6)	30.5 (28.0; 88.0)	33.5 (29.3; 41.3)
**Cholesterol (mg)**	271.5 (118.3; 1157.0)	341.4 (169.8; 1,054.1)	352.1 (93.0; 652.7)
**Fiber (g)**	20.0 ± 5.2	10.5 ± 2.3	23.3 ± 5.2

SAH: Systemic arterial hypertension; DM: Diabetes mellitus; SFA: Saturated fatty acids; PUFA: Polyunsaturated fatty acids; MUFA: Monounsaturated fatty acids.

Numbers are as: Mean ± Standard Error or Median (minimum;maximum).

p < 0.05 (Tukey HSD or Dunnett post hoc tests):

* CGL *vs*. EUT;

^#^ CGL *vs*. OB;

^&^ EUT *vs*. OB.

In the test meal, no significant differences were found in caloric and macronutrients intakes. However, the CGL group had lower satiation and satiety times than those of the EUT group and the OB group had lower satiation time than that of the EUT group (Table 3).

**Table 3:** Appetite scores of the test meal and visual analogue scale (VAS) features of CGL patients, obese (OB) and eutrophic subjects (EUT), before (T0) and 90 minutes after meal ingestion (T90).

		EUT	OB	CGL
**Test Meal**				
Energy intake (kcal)		598.7 (230.1; 1452.2) (n = 10)	541.8 (168.3; 736.6) (n = 10)	764.7 (427.0; 1187.0) (n = 10)
Carbohydrates intake (kcal)		399.1 ± 78.5 (n = 10)	328.5 ± 34.6 (n = 10)	483.0 ± 46.3 (n = 10)
Protein intake (kcal)		86.6 ± 19.5 (n = 10)	75.0 ± 17.5 (n = 10)	101.9 ± 10.4 (n = 10)
Lipid intake (kcal)		112.7 (53.8; 434.8) (n = 10)	113.8 ± 21.0 (n = 10)	151.3 (90.6; 256.0) (n = 10)
Satiation time (seconds)		720.0 (360.0; 1,260.0) (n = 10)	450.0 (300.0; 720.0)^&^ (n = 10)	420.0 (180.0; 1,140.0)* (n = 10)
Satiety time (seconds)		12,768.0 ± 762.0 (n = 10)	10,932.0 ± 960.0 (n = 10)	8,496.0 ± 1,122.0* (n = 10)
**Visual Analogue Scale**				
Hunger	T0	69.0 (26.5; 91.0)	55.0 (33.5; 94.0)	32.0 (5.0; 95.5)
	T90	10.0 (0.0; 90.0)	50.0 (14.0; 95.0) ^&^	39.5 (5.0; 100.0)
	Δ	-45.6 ± 8.7 (n = 9)	-10.6 ± 8.4 (n = 9)	-3.8 ± 14.0* (n = 11)
Fullness	T0	14.0 (7.0; 29.5)	15.7 (6.5; 50.0)	7.0 (0.0; 80.0)
	T90	68.8 ± 10.4	53.2 ± 8.9	43.1 ± 11.5
	Δ	53.0 ± 9.8 (n = 9)	37.5 ± 7.2 (n = 8)	25.8 ± 15.0 (n = 11)
Satiety	T0	19.3 ± 3.8	22.7 ± 4.6	18.2 ± 6.8
	T90	56.4 ± 10.3	47.2 ± 9.8	45.4 ± 10.8
	Δ	37.2 ± 11.1 (n = 9)	28.4 ± 7.8 (n = 8)	27.2 ± 14.4 (n = 11)
Prospective food consumption	T0	77.0 (49.0; 100.0)	72.0 (50.0; 95.0)	90.5 (20.0; 98.0)
	T90	21.0 (11.5; 91.0)	33.5 (10.0; 100.0)	43.5 (4.0; 86.0)
	Δ	- 40.0 ± 9.6 (n = 9)	-29.8 ± 9.8 (n = 9)	-24.0 ± 15.3 (n = 11)
Desire to eat	T0	55.0 ± 6.4	48.7 ± 6.2	64.5 ± 7.5
	T90	68.1 ± 8.3	47.3 ± 7.9	66.0 ± 6.0
	Δ	15.8 (-37.3; 68.7) (n = 9)	5.9 (-80.0; 32.5) (n = 9)	7.1 (-42.4; 22.1) (n = 11)
Appetite score	T0	273.9 (216.5; 323.5)	258.4 (239.3; 287.5)	265.9 (179.3; 359.2)
	T90	178.4 ± 18.0	201.5 ± 15.0	232.3 ± 22.8
	Δ	-95.4 ± 20.5 (n = 9)	-56.9 ± 16.4 (n = 9)	-34.1 ± 27.8 (n = 11)

Numbers are as: Mean ± Standard Error or Median (minimum;maximum).

p < 0.05 (Tukey HSD or Dunnett post hoc tests):

* CGL *vs*. EUT;

^#^ CGL *vs*. OB;

^&^ EUT *vs*. OB.

The VAS test showed that the CGL group had a significantly lower attenuation of hunger after the test meal than the EUT group (Table 3).

Although all CGL patients were under the treatment with fibrates for hypertriglyceridemia, their levels of triglycerides were increased. Also, very low density lipoprotein cholesterol (VLDL-c) levels were higher while those of high density lipoprotein cholesterol (HDL-c) were lower in the CGL group than in both the EUT and the OB groups, at both T0 and T90. Low density lipoprotein cholesterol (LDL-c) levels were lower in CGL than EUT at T0 and EUT and OB at T90. The insulin levels of the CGL individuals were significantly higher than those of the EUT group at both times. The OB group had significantly higher insulin levels than the EUT group at T90. The homeostasis model assessment insulin resistance index (HOMA-IR) of the CGL group was significantly higher than that of the EUT group and tended to be so also in comparison to the OB group (p = 0.08). The fact that all patients were receiving metformin for diabetes control emphasizes the severity of their metabolic derangements (Table 4).

**Table 4:** Serum parameters and HOMA indexes of CGL patients, obese (OB) and eutrophic subjects (EUT).

		EUT	OB	CGL
Tryglicerides (mg/dL)	T0	64.0 (23.0; 107.0) (n = 10)	130.5 (40.0; 221.0) (n = 10)	265.5 (97.0; 833.0)*^#^ (n = 10)
	T90	97.0 (30.0; 145.0) (n = 10)	181.5 (41.0; 250.0) (n = 10)	336.0 (194.0; 876.0)*^#^ (n = 11)
Total cholesterol (mg/dL)	T0	165.0 (123.0; 187.0) (n = 10)	169.5 (125.0; 296.0) (n = 10)	148.5 (103.0; 281.0) (n = 10)
	T90	156.9 ± 8.5 (n = 10)	177.9 ± 16.2 (n = 10)	169.7 ± 16.6 (n = 11)
HDL-c (mg/dL)	T0	42.3 ± 3.1 (n = 10)	43.5 ± 3.7 (n = 10)	28.9 ± 2.7*^#^ (n = 10)
	T90	41.6 ± 2.9 (n = 10)	42.2 ± 3.4 (n = 10)	27.1 ± 2.5*^#^ (n = 11)
LDL-c (mg/dL)	T0	102.7 ± 6.7 (n = 9)	87.2 ± 5.7 (n = 10)	66.9 ± 10.4* (n = 10)
	T90	97.8 ± 7.7 (n = 10)	86.7 ± 7.0 (n = 10)	56.9 ± 9.0*^#^ (n = 11)
VLDL-c (mg/dL)	T0	12.8 (4.6; 21.4) (n = 9)	26.1 (8.0; 44.2) (n = 10)	53.1 (19.4; 166.6)*^#^ (n = 10)
	T90	19.4 (6.0; 29.0) (n = 10)	36.3 (8.2; 50.0) (n = 10)	67.2 (38.8; 175.2)*^#^ (n = 11)
Glucose (mg/dL)	T0	85.0 (66.0; 98.0) (n = 10)	86.5 (73.0; 95.0) (n = 10)	94.0 (72.0; 334.0) (n = 10)
	T90	86.5 (66.0; 129.0) (n = 10)	101.5 (70.0; 127.0) (n = 10)	200.0 (56.0; 407.0)*^#^ (n = 11)
Insulin (UI/mL)	T0	3.1 (0.5; 9.0) (n = 9)	6.3 (2.3; 9.8) (n = 10)	16.8 (2.8; 31.0)* (n = 8)
	T90	16.6 (7.0; 28.5) (n = 9)	41.9 (15.7; 98.7)^&^ (n = 10)	30.7 (5.0; 104.5)* (n = 11)
HOMA-IR	T0	0.7 (0.1; 1.2) (n = 9)	1.3 (0.5; 2.1)(n = 10)	4.1 (0.6; 18.8)* (n = 8)
HOMA-β	T0	75.4 ± 22.4 (n = 9)	110.0 ± 14.9 (n = 10)	178.3 ± 63.0 (n = 8)

HDL-c: High-density lipoprotein cholesterol; LDL-c: ow-density lipoprotein cholesterol; VLDL-c: Very low-density lipoprotein cholesterol; HOMA-IR: Homeostasis model assessment insulin resistance; HOMA- β: Homeostasis model assessment β-cell function.

Numbers are as: Mean ± Standard Error or Median (minimum;maximum).

p < 0.05 (Tukey HSD or Dunnett post hoc tests):

* CGL *vs*. EUT;

^#^ CGL *vs*. OB;

^&^ EUT *vs*. OB.

Serum levels of leptin were lower (p = 0.08) in the CGL group and higher in the OB group than in the EUT group. Adiponectin levels were lower in the CGL group in comparison to both the EUT and the OB groups, at both times (Table 5).

**Table 5:** Serum cytokines of CGL patients, obese (OB) and eutrophic subjects (EUT).

		EUT	OB	CGL
PAI-1 (ng/mL)	T0	4.1 (1.2; 8.3) (n = 10)	3.9 (2.6; 20.5) (n = 10)	4.6 (1.3; 8.9) (n = 10)
	T90	3.9 (2.0; 7.6) (n = 10)	4.0 (2.9; 20.4) (n = 9)	4.0 (0.1; 7.5) (n = 11)
Leptin (ng/mL)	T0	12.0 (0.4; 17.1) (n = 9)	31.9 (11.3; 102.0)^&^ (n = 10)	1.0 (0.2; 3.6)^#^ (n = 10)
	T90	8.8 (0.8; 17.5) (n = 10)	26.8 (10.0; 54.6)^&^ (n = 9)	3.0 (0.0; 6.8)^#^ (n = 9)
Adiponectin (μg/mL)	T0	5.36 (3.64; 8.70) (n = 10)	3.75 (0.48; 7.48) (n = 10)	0.31 (0.04; 6.80)*^#^ (n = 10)
	T90	5.52 (3.29; 11.58) (n = 10)	4.60 (0.32; 7.49) (n = 10)	0.44 (0.16; 7.13)*^#^ (n = 10)

PAI-1: Plasminogen activator inhibitor-1.

Numbers are as: Mean ± Standard Error or Median (minimum;maximum).

p < 0.05 (Tukey HSD or Dunnett post hoc tests):

* CGL *vs*. EUT;

^#^ CGL *vs*. OB;

^&^ EUT *vs*. OB.

**Fig 1:**
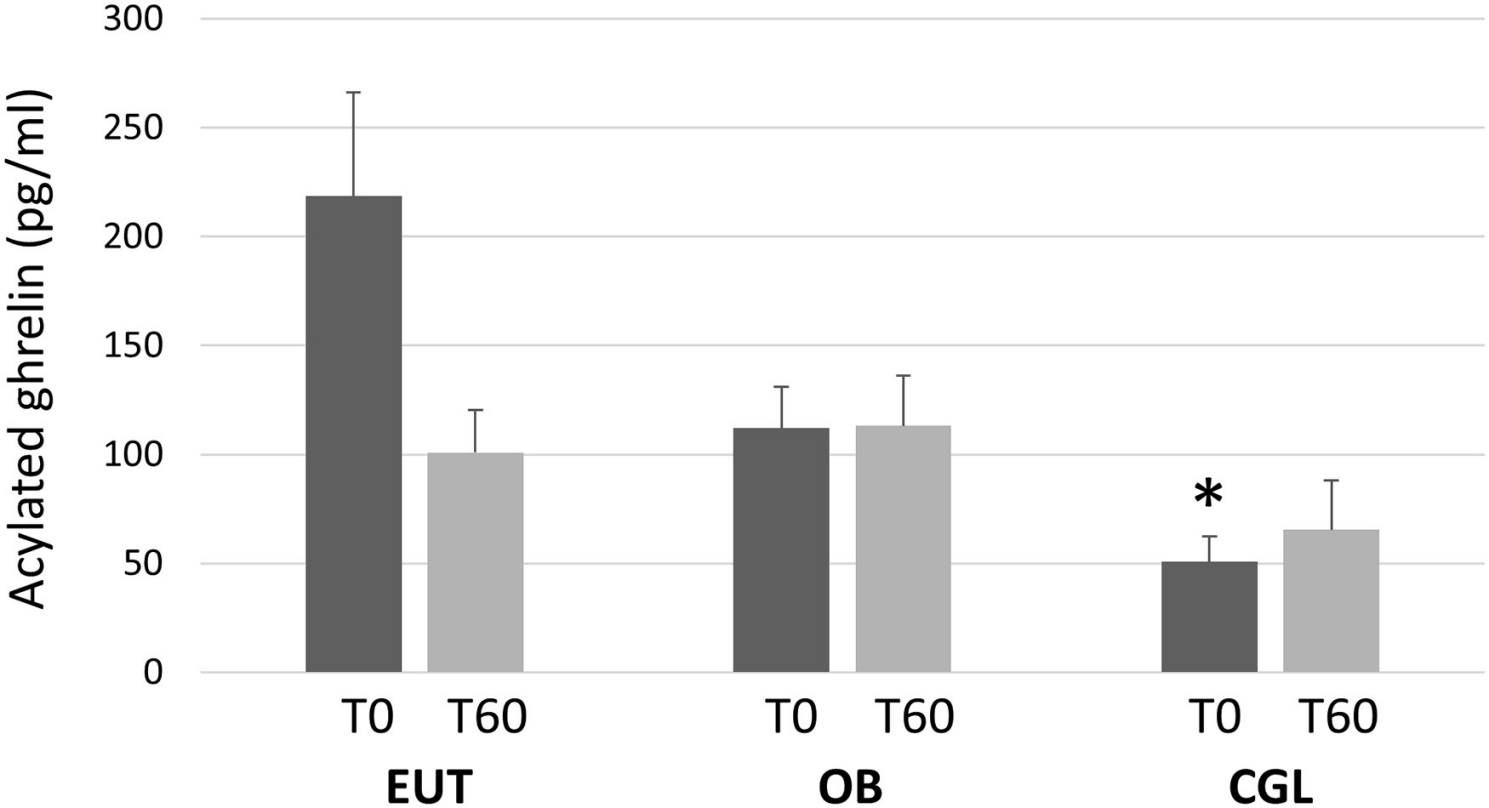
Plasma levels of acylated ghrelin, before (T0) and 60 minutes after the intake of the test meal (T60) in eutrophic (EUT, N = 8), obese (OB, N = 6) and patients with congenital generalized lipodystrophy (CGL, N = 8). (Tukey HSD post hoc): * *vs*. EUT.

Acylated ghrelin levels were evaluated at T0 and T60 and the results are presented in Fig 1. At T0, the levels were significantly lower in the CGL group than in the EUT group (p = 0.03) and showed a trend in relation to the OB group (p = 0.06) F(_2,19_) = 7.568, p = 0.004). In the EUT group, mean ghrelin levels fell after meal intake, being lower at T60 than at T0 (p = 0.053). Differently, no trend to declining ghrelin levels at T60 was observed in both the OB (p = 0.566) and the CGL (p = 0.607) groups.

## Discussion

The present findings show that the caloric intake of the CGL group was similar to that of the EUT and OB groups, as assessed both by the food record and during the *ad libitum* meal test. Since the early papers describing the CGL disease [22], these patients have been described as having voracious appetite [8]. However, after a careful examination of the available data we were not able to find actual comparisons of caloric intake between CGL patients and healthy individuals, as performed in the present study.

The analysis of food records of a group of Brazilian CGL patients has shown a daily intake of around 4,000kcal. Although this value can be considered high in comparison to the calculated dietary reference intake (DRI) of the patients (around 2,600 kcal/day), the authors did not include eutrophic individuals in their study [33].

The very low leptinemia reported for CGL patients [34,35] has been confirmed in the present study. Several studies examined the effect of recombinant leptin treatment in groups of lipodystrophic individual that included CGL patients. They found decreased caloric intake, although the pre-treatment intake was either of around 2,000 kcal [24] or not reported [36], as well as decreased hunger and increased satiety [24–26].

Increased basal metabolic rate as a cause for hyperphagia has been proposed in CGL patients rate [37], while a study has shown that, although high in relation to total body weight, they had normal energy expenditure and resting metabolic rate in relation to the lean body mass [19].

In the present study, we did demonstrate significantly shorter satiation and satiety times in the CGL than in the EUT group and that the meal was not able to attenuate hunger in the CGL group. Importantly, both the CGL and the OB individuals reached satiation in a very short time having ingested a similar amount of calories as the EUT subjects. It is interesting that, although the satiation time was similar between the CGL and the OB groups, only the former presented a reduced satiety time. Moreover, the appetite scores measured before and after the meal showed the smallest variation in the CGL group. A study evaluating the effect of a recombinant leptin treatment in lipodystrophic individuals has reported that the hormone decreased satiation and increased satiety times while it decreased the desire to eat score [24].

The lipid profile findings of this study showed dyslipidemia in CGL group, characterized by hypertriglyceridemia and low HDL levels. These findings corroborated previous reports [9,36,38] and have been attributed to the incapacity of storing dietary triglycerides in the adipose tissue [7]. It is of note that these parameters were also significantly altered in relation to those of the OB group.

The LDL levels were significantly lower while those of VLDL were higher in the CGL group than in the EUT group. There are few studies addressing LDL levels in CGL subjects, having reported either normal levels [9] or decreasing of levels after 3 years of leptin treatment [39]. The increased VLDL levels agree with previous reports in CGL individuals [40]. One study in mice bearing the type 4 CGL (*CAV-1* mutation) found increased levels of non-HDL cholesterol [41]. High VLDL secretion in the presence lipid storage defects have been associated with liver *de novo* lipogenesis [42,43]. In diabetic patients, a study related VLDL secretion to the fat content in liver [44]. Interestingly, the ingestion of the meal did not affect the lipid profile alterations seen in the fasting state. One study in lipodystrophic patients with metabolic syndrome argued that their insulin resistance could reduce HDL production and increase its metabolism while high VLDL levels could be related to low lipoprotein lipase levels [45].

The evaluation of fasting glucose and insulin levels indicated the presence of insulin resistance, as insulinemia was found to be very high, in agreement with other authors evaluating CGL patients [9,46]. The HOMA-IR index confirmed the presence of deranged glucose homeostasis. Importantly, 90 minutes after the meal, the CGL group still presented higher glycemia than both the EUT and OB groups and higher insulinemia than the EUT group. It is relevant to point out that 8 out of the 11 patients were diabetic and were in use of metformin, unlike the OB individuals. Nevertheless, the HOMA-IR values indicated that the CGL individuals tended to be more insulin-resistant than the OB group. The insulin resistance present in CGL has been attributed to both the ectopic fat accumulation in the liver and to the low adiponectin levels due to the lack of mature adipocytes in the adipose tissue [47].

The low levels of leptin and adiponectin of the CGL group corroborate other reports [9,48]. Because adiponectin favors insulin sensitivity [49], its low production is relevant to the insulin resistance of the CGL [7,48]. Studies comparing different types of lipodystrophy found that both leptin and adiponectin levels are particularly low in the generalized forms, with even lower levels found in the congenital than in the acquired generalized variants [48,50,51]. In CGL, adiponectin levels have been found to be lower in type 1 (*AGPAT2* mutation) than in type 2 (*BSCL2* mutation) [52,53]. This would increase appetite and contribute to hypertriglyceridemia in CGL patients [7].

PAI-1 is a cytokine produced by the adipose tissue that exerts important metabolic and vascular actions. High PAI-1 levels have been shown to induce insulin resistance and to be a predictor of cardiovascular risk [54]. We did not find any alterations in PAI-1 levels in the CGL group. We couldn’t find previous reports on this adipokine in CGL patients while levels were found to be either increased [55] or unchanged [56] in the lipodystrophy induced by anti-HIV drugs.

The fasting levels of acylated ghrelin were lower in the CGL group than in the EUT group. After the meal, ghrelin levels tended to decrease in the EUT individuals while no such trend was observed in the CGL and OB subjects. We found only one study comparing ghrelin responses to food intake between CGL and normal individuals. Those authors measured only total ghrelin and found similar results as those reported here for acylated ghrelin, i.e., no post-meal decrease [19].

Ghrelin is a hormone produced when the stomach is empty and acts stimulating hunger. The enzyme ghrelin-O-acyltransferase is responsible for the conversion of des-acyl ghrelin to its acylated form, which is able to stimulate hypothalamic receptors located mainly in the arcuate nucleus [15,57,58]. The physiological role of ghrelin as an appetite stimulator has been demonstrated in both animals and humans. Plasma levels of both total and acylated ghrelin have been shown to increase during fasting while they decrease after a meal, as part of the satiation signaling in normal subjects [59,60]. Moreover, a role for ghrelin as a long-term controller of body weight has been proposed based on the observation that diet-induced weight loss increased ghrelin levels in obese subjects [21,61]. Fasting ghrelin has been shown to be low in obesity, while post-prandial levels have been shown to be reduced in some [20,21] but not all studies [62]. The human adipose tissue has been shown to express the enzyme ghrelin O-acyltransferase and to synthesize ghrelin [63]. Although this could be relevant to explain, at least in part, the lower levels of acylated ghrelin in the CGL patients, this suggestion does not apply to the obese individuals.

Studies addressing the existence of an effect of leptin on the ghrelin system have yielded conflicting results, as leptin levels have been found to correlate positively, negatively, or not at all with ghrelin levels in obese and lean individuals [64]. A study in CGL patients have found that the treatment with recombinant leptin for 4 months reduced plasma levels of total ghrelin [24].

In contrast, studies performed in overweight non-diabetic subjects [65] and primary culture of rat gastric cells [66] have provided evidence that insulin may be a negative regulator of ghrelin levels. Additionally, in hyperinsulinemic conditions, such as obesity, insulin has been attributed a role in attenuating the postprandial drop in ghrelin levels [16,21]. These data agree with the present results in the OB group, showing that fasting levels and the response of acylated ghrelin to the test meal were similar to those of the CGL patients. Our present results suggest that the very high levels of insulin present in the CGL group have been relevant in determining the low levels of ghrelin and the absence of the expected fall after the meal.

In conclusion, the present data corroborated the findings of hypertriglyceridemia, hypoleptinemia, and insulin resistance in CGL patients. We found that their dyslipidemia and insulin resistance were even more pronounced than those of obese non-diabetic subjects. Although the caloric intake of the CGL patients was similar to that of the eutrophic controls, their satiation and satiety times were decreased and the meal failed to attenuate their hunger sensation. Importantly, ours is the first report of acylated ghrelin levels in this rare disease. We were able to demonstrate that its levels were inappropriately low at the fasted state and that the expected drop after the meal was absent. This indicates that the very short satiation time shown by the CGL patients was probably not influenced by ghrelin. It is possible that, in view of the extreme hypoleptinemia and insulin resistance, other orexigenic factor, such as neuropeptide Y, were responsible for this alteration. On the other hand, the lack of the physiological decline after food intake may have influenced the low satiety time and the small attenuation of the hunger sensation.

## Supporting information

S1 Table Prior food records of CGL patients. (XLSX)

S2 Table General characteristics of CGL patients. (XLSX)
